# Sensitive Detection of α-Conotoxin GI in Human Plasma Using a Solid-Phase Extraction Column and LC-MS/MS

**DOI:** 10.3390/toxins9080235

**Published:** 2017-07-28

**Authors:** Shuo Yu, Bo Yang, Liangping Yan, Qiuyun Dai

**Affiliations:** 1 Beijing Institute of Biotechnology, Beijing 100071, China; o_yys@163.com; 2 Zhonglian (Beijing) Pharmaceutical Technology Co. Ltd., Beijing 101111, China; bo.yang@zlapharma.com (B.Y.); liangping.yan@zlapharma.com (L.Y.)

**Keywords:** α-conotoxin GI, detection, solid-phase extraction, LC-ESI-MS, human plasma

## Abstract

α-conotoxin GI, a short peptide toxin in the venom of *Conus geographus*, is composed of 13 amino acids and two disulfide bonds. It is the most toxic component of *Conus geographus* venom with estimated lethal doses of 0.029–0.038 mg/kg for humans. There is currently no reported analytical method for this toxin. In the present study, a sensitive detection method was developed to quantify GI in human plasma using a solid-phase extraction (SPE) column (polystyrene–divinyl benzene copolymer) combined with liquid chromatography/electrospray ionization tandem mass spectrometry (LC-ESI-MS/MS) in the multiple reaction monitoring (MRM) mode. The plasma samples were treated with a protein precipitating solvent (methanol: acetonitrile = 50:50, *v*/*v*). GI in the solvent was efficiently extracted with an SPE column and was further separated by a Grace Alltima HP C_18_ (50 × 2.1 mm, 5 μm) column at a flow rate of 0.4 mL/min. Water (with 2% methanol) acetonitrile (with 0.1% acetic acid) was selected as the mobile phase combination used in a linear gradient system. α-Conotoxin GI was analyzed by an API 4000 triple quadrupole mass spectrometer. In the method validation, the linear calibration curve in the range of 2.0 to 300.0 ng/mL had correlation coefficients (*r*) above 0.996. The recovery was 57.6–66.8% for GI and the internal standard. The lower limit of quantification (LLOQ) was 2 ng/mL. The intra- and inter-batch precisions were below 6.31% and 8.61%, respectively, and the accuracies were all within acceptance. GI was stable in a bench-top autosampler through long-term storage and freeze/thaw cycles. Therefore, this method is specific, sensitive and reliable for quantitative analysis of α-conotoxin GI in human plasma.

## 1. Introduction

Commonly known as cone snails, the marine gastropod genus *Conus* is a hyperdiverse group of specialized predators, which use venom to subdue the prey and for self-defense. There are an estimated number of more than 700 species of cone snails around the world [1]. Generally, cone snails are divided into three groups based on the prey they subdue: piscivorous, molluscivorous and vermivorous [2,3]. The vermivorous species are predominant and account for about 75% of all cone snails [4], but the piscivorous (~10%) species are the most poisonous and some even fatal to humans [5,6,7]. Among all piscivorous species, *Conus geographus* (*C. geographus*) is the most dangerous to humans, which resulted in half of the known human envenomations and almost all were fatal [8,9]. The estimated lethal dose of the *C. geographus* venom is about 0.029 mg/kg~0.038 mg/kg for humans [10]. After being stung by this species, people experience numbness and local swelling at the sting site, followed by a series of toxic symptoms, including blurred vision or diplopia, fatigue, nausea, stomach cramps, facial paralysis, etc. [7]. Symptoms can aggravate over time leading to generalized paralysis and respiratory failure. Without medical treatment, coma and death may follow.

**Figure 1:**
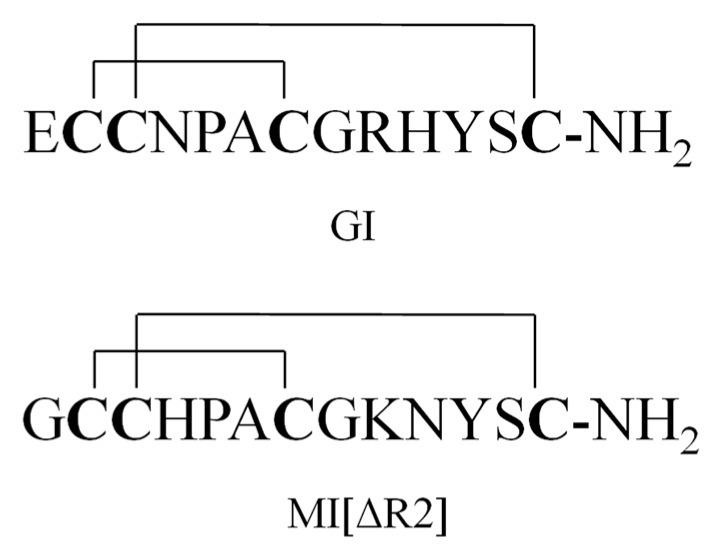
Structure of GI and MI[ΔR2].

A series of paralytic peptides have so far been found in the venom of *C. geographus* [11]. These peptide conotoxins selectively target ion channels, e.g., Na^+^, K^+^ and Ca^2+^ channels, or membrane receptors (nAChR, NMDAR and G-protein-coupled receptors) [12,13,14,15]. For example, α-conotoxins GI, GIA and GII are potent antagonists for nicotine acetylcholine receptors (nAChRs) [16,17,18]; μ-conotoxins GIIIA, GIIIB and GIIIC selectively target sodium ion channels [19,20,21]; ω-conotoxins GVIA, GVIB, GVIC, GVIIA and GVIIB inhibit calcium ion channels [22,23,24]. GI (Figure 1) contains 13 amino acid residues and two disulfide bridges and is the most poisonous of all the peptide toxins in the venom of *C. geographus*. It functions by selectively inhibiting muscular nAChRs. The lethal dose of GI in mice is between 8 and 12 μg/kg (intraperitoneal, i.p.) [25,26]. Moreover, no antidote or anti-venom is currently available for GI. Due to of its ease of production, GI may be potentially used by terrorists as a biological weapon [27].

In the present study, a sensitive analytical method for GI in human blood plasma was developed. GI was sensitively detected using a solid-phase extraction (SPE) column combined with liquid chromatography/electrospray ionization tandem mass spectrometry (LC-ESI-MS/MS) in the multiple reaction monitoring (MRM) mode. After precipitation with the mixed solvents of methanol and acetonitrile, GI in human plasma was efficiently concentrated with an SPE column and was further separated by a reverse-phase column. The analytical method was validated for linearity, accuracy, precision, lower limit of quantification (LLOQ), and stability. The recovery rate of GI in blood samples was also determined. The results demonstrate that this method can achieve quick and sensitive determination of GI in human plasma and blood in envenomation accidents and potential bioterrorism incidents.

## 2. Results

### 2.1. Sample Treatment

The plasma samples of GI were treated with methanol/acetonitrile (50:50, *v*/*v*) for protein precipitation. The percent recoveries of GI and internal standard (IS) MI[ΔR2] (Figure 1) were better than those obtained by using acetonitrile alone (data not shown). After the dilution of the above extract with water (the final ratio of solvent to water was 1:3), the GI samples were further concentrated and purified by an SPE column with methanol/water. GI was eluted with 70% methanol/water containing 1% acetic acid and collected after washing with 10% and 40% methanol.

### 2.2. Liquid Chromatography

**Figure 2:**
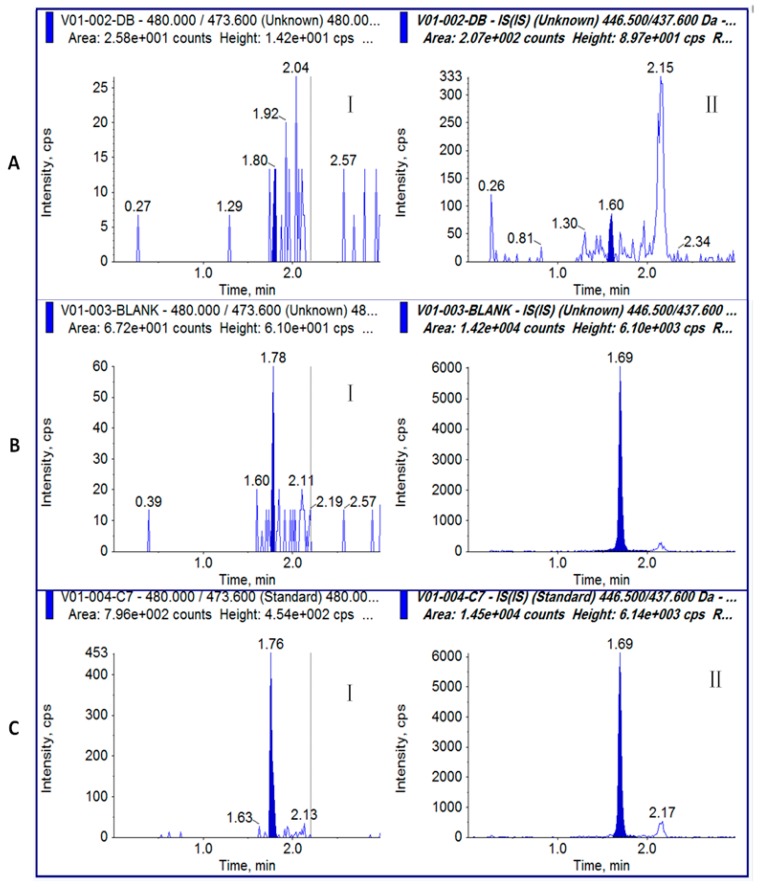
Representative chromatograms for toxin samples: (**A**) plasma blank without MI[ΔR2]; (**B**) plasma blank with MI[ΔR2]; (**C**) GI (I) and IS (MI[ΔR2]) (II) at LLOQ (2 ng/mL).

GI and MI[ΔR2] were separated on a C_18_ reverse phase column with optimized elution conditions from 0.2 min to 1.3 min by adjusting the ratio of mobile phases A and B. The retention time was 1.76 min for GI and 1.69 min for IS (MI[ΔR2]) (Figure 2) under the optimized elution conditions (Section 5.3).

### 2.3. MS/MS

**Figure 3:**
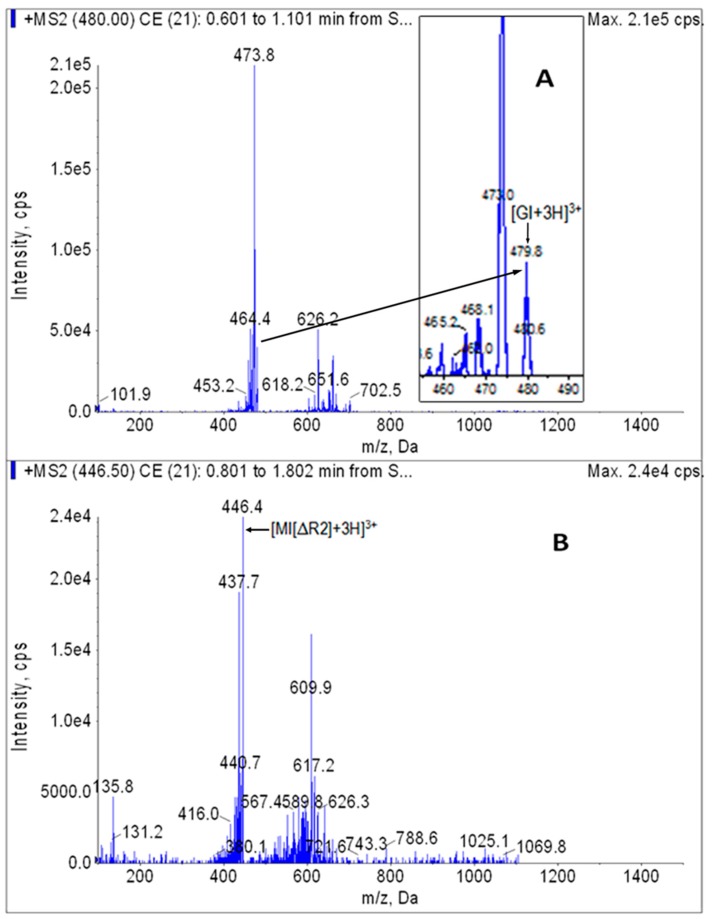
[M + H]^+^ MS/MS spectrograms of GI (**A**) and internal standard MI[ΔR2] (**B**).

Stock solution of GI or internal standard MI[ΔR2] was diluted with the diluent and injected in the peristaltic pump mode. The optimized parameters for the MRM analysis of analytes (GI) and internal standard (MI[ΔR2]) were 480.0/473.6 and 446.5/437.6, respectively. In addition, transition 480.0→626.6 was also used to avoid false positives in the GI analysis. The MS/MS full scan spectrogram is shown in Figure 3.

### 2.4. Specificity

No significant interfering peaks were observed in the chromatograms of the six plasma blanks at the retention times of GI and the IS (MI[ΔR2]) (Figure 2A). The responses of interferences at the retention time of GI and MI[ΔR2] were lower than 20% and 5% of the LLOQ responses of GI and MI[ΔR2] (Figure 2), respectively.

### 2.5. Linearity and Sensitivity

**Figure 4:**
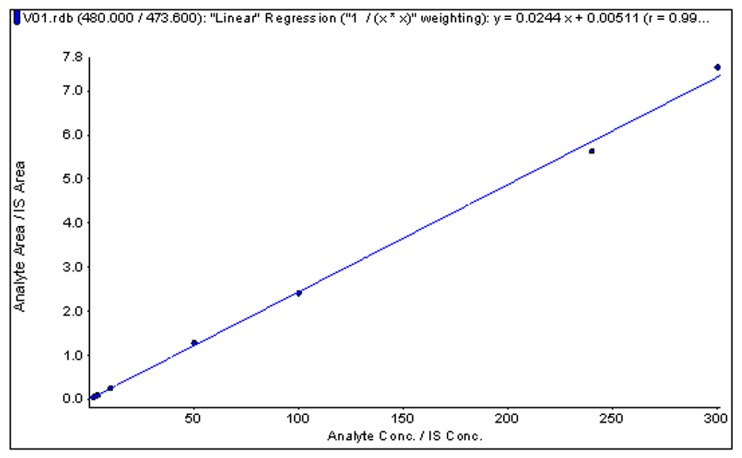
Typical standard curve of α-conotoxin GI in human plasma.

The response was linear over the tested concentration range of 2.0–300.0 ng/mL. The correlation coefficient (*r*) of six batches was 0.996~0.999, which met the acceptance criteria of no less than 0.99. A typical calibration curve is shown in Figure 4. The LLOQ of GI was 2 ng/mL. The precision (RSD%) and accuracy (RE%) at the LLOQ were 12.70% and −7.27% (*n* = 6, Table 1), respectively.

### 2.6. Assay Precision and Accuracy

The inter-batch precision and accuracy of calibration standards were evaluated for seven different runs. The precision (RSD%) ranged from 1.31 to 8.83%, while the accuracy (RE%) ranged from −4.28 to 5.21% (*n* = 7). The intra-and inter-batch precision and accuracy of the QC samples were also evaluated (Table 2). The intra-batch precision (RSD%) ranged from 4.08 to 6.31% with an accuracy range of −4.80 to 1.25% (*n* = 6). The inter-batch precision (RSD%) ranged from 7.02 to 8.61% with an accuracy (RE%) range of 1.27 to 1.50% (*n* = 7). These results indicate that the method is accurate, reliable and reproducible.

### 2.7. Matrix Effect and Recovery

The spike recoveries of GI at low, mid, and high levels (6.0, 40.0, 225.0 ng/mL) were 99.10 ± 6.83%, 111.96 ± 3.06% and 113.32 ± 7.66% (*n* = 6), respectively, with a mean of 108.12%. The mean spike recovery of MI[ΔR2] was 89.36 ± 2.36% (*n* = 6). Therefore, a minimal matrix effect was observed for GI and MI[ΔR2]. The extraction recoveries of GI QC samples were 52.81 ± 2.53%, 61.07 ± 9.19% and 58.88 ± 3.90% (*n* = 6), respectively. The mean recoveries of GI and MI[ΔR2] were 57.59% and 66.76 ± 2.36% (*n* = 6), respectively. The results are summarized in Table 3.

### 2.8. Stability

The stabilities of benchtop, freezer storage and freeze/thaw cycles of the QC samples were evaluated. The results are summarized in Table 4. GI was stable after three freeze (−20 °C) and thaw cycles in plasma. It was also stable in the plasma for at least 5.25 h at room temperature and for 14 days at −20 °C. Stock solutions of GI and internal standard MI[ΔR2] were found to be stable for 19 days at 2–8 °C and for 6 h at room temperature.

### 2.9. Analysis of GI in Human Blood

As shown in Table 5, the relative recovery of GI in human blood was 73.48% ± 9.55% (*n* = 4), and the absolute recovery of GI in human blood was 42.32% ± 5.50% (*n* = 4). GI in human blood was stable for 3 h at room temperature. The mean ratio of GI samples at room temperature for 3 h to GI samples at 0 h was 103.54 ± 13.09% (*n* = 4).

## 3. Discussion

Generally, a precipitating solvent is used to precipitate most proteins in plasma and keep the analytes in solution [24,28,29]. In the present assay, a mixed solvent of methanol/acetonitrile (50:50, *v*/*v*) was used. α-Conotoxin GI and internal standard MI[ΔR2] were soluble upon addition of the mixed solvent to the plasma samples. This method is similar to the analyses of small compounds, but different from the analyses of longer ^125-^I-peptides, in which the analyte is also precipitated together with plasma proteins.

Several columns have been used to enrich analytes so far, such as the C_18_ reverse phase column, ion exchange column and magnetic beads [30,31,32,33]. In this experiment, the polystyrene–divinyl benzene SPE column was used to recover α-conotoxin GI in the precipitating solvent. Compared to other SPE columns, the polystyrene–divinyl benzene column was less expensive, easier to use, and the recovery (>52.8%) was satisfactory. It is possible to improve the recovery by increasing the volume of elution solvent. However, the interfering substances would also increase, resulting in matrix effects.

α-conotoxin MI, another α-conotoxin found in *C. magus*, is also fatal to human [26]. In order to obtain the internal standard where the molecular weight is close to GI and contains 13 amino acids with two disulfide bonds, MI mutant (MI[ΔR2]) was selected as an internal standard.

Furthermore, our results demonstrated that GI was stable in the plasma for at least 5.25 h at room temperature and for 14 days at −20 °C; it was also stable in human blood for at least 3 h at room temperature. Accounting for the absolute recovery of conotoxin GI in human blood (42%) and the LLOQ of GI (2 ng/mL) in human plasma, this analytical method can detect GI in the blood as low as 5 ng/mL. The lethal dose of GI (intravenous, i.v.) in human has not been established so far. However, the lethal dose of GI in mice (i.p.) has been determined to be 8~12 μg/kg [25,26], suggesting a possible LD_50_ of more than 8 μg/kg (~8 ng/mL, i.v.) for humans. Therefore, this method is able to detect GI in at least 3 h after intoxication, requiring approximately 2 ml of the blood of the poisoned victim.

## 4. Conclusions

In this study, we developed an SPE-LC-ESI-MS method for the detection of α-conotoxin GI in human plasma. GI and internal standard MI[ΔR2] were well recovered from human plasma after protein precipitation with the mixed solvent of methanol/acetonitrile (50/50%, *v*/*v*) and an SPE column (polystyrene–divinyl benzene copolymer). This method is sensitive with the LLOQ of 2.0 ng/mL and has good linearity (*r* > 0.996) in the concentration range of 2.0–300.0 ng/mL. The intra- and inter-batch precisions were below 6.31% and 8.61%, respectively, and the accuracies were all within acceptance. GI in the plasma could be sensitively detected after storage in the plasma at room temperature for at least 5.25 h and at −20 °C for 14 days. Furthermore, this method was successfully applied to the quantification of GI in human blood. It may be valuable for the identification of envenomation accidents and possible bioterrorism incidents.

## 5. Experimental Section

### 5.1. Chemicals and Reagents

α-conotoxin GI (purity > 98%) and α-conotoxin MI mutant MI[ΔR2](internal standard, IS) (purity > 98%) were synthesized using a previously described method [34]. Drug-free human plasma and full blood (with sodium citrate as anticoagulant) were collected from volunteers and outdated transfusion blood obtained from the 307th Hospital of Chinese People’s Liberation Army (Beijing, China). All solvents were of HPLC grade. Acetonitrile and methanol were purchased from Honeywell, Morris Plains, NJ, USA. Formic acid and acetic acid were purchased from Dikma, Lake Forest, CA, USA. Deionized water (≥18.3 MΩ) was produced by a nanopure water purification system (Thermo scientific, Waltham, MA, USA). Solid-phase extraction (SPE) columns (polystyrene-divinyl benzene copolymer) (30 mg × 1 mL, 50 μm, 80 A) were purchased from Tianxingda Technology Co. Ltd. (Tianjin, China).

### 5.2. Preparation of Standards

A diluent of 0.2% formic acid (*v*/*v*) was prepared in deionized water. Stock solutions of GI (1.00 mg/mL) and internal standard MI[ΔR2] (1.00 mg/mL) were prepared in the diluent. The working solutions of GI were prepared at concentrations of 20.0, 40.0, 100.0, 500.0, 1000.0, 2400.0, 3000.0, 10,000.0 and 100,000.0 ng/mL by diluting aliquots of the stock solution with the diluent. The QC working solutions of GI were prepared at concentrations of 60.0, 400.0, 2250.0, 10,000.0 and 100,000.0 ng/mL by spiking aliquots of the QC stock solution (1.0 mg/mL) into the diluent. QC standards of 6.0, 40.0 and 225.0 ng/mL were prepared by diluting 30 µL of the corresponding QC working solutions into 300 µL drug-free human plasma. All stock solutions were stored at 4 °C before analysis. All working solutions were freshly prepared before daily use.

### 5.3. Sample Preparation and Extraction Procedure

A 0.30 mL aliquot of human drug-free plasma sample was spiked with 30 μL of the working solution of GI or QC. Unknown samples or blank control samples were spiked with 30 μL of the diluent. The internal standard solution (30 μL) was then spiked into each sample and fully mixed, followed by the addition of 0.60 mL precipitating agent (methanol:acetonitrile = 50:50, *v*/*v*). The solution was vortexed and centrifuged at 12,000 rpm for 5 min. A 0.750 mL aliquot of the supernatant in each sample was mixed with 2.250 mL water. The resulting solution was extracted on the SPE column. The SPE column was rinsed with methanol (0.500 mL) twice, and conditioned twice with pure water (0.500 mL) prior to use. The prepared sample (3.0 mL) was then transferred onto the SPE column, and washed by 0.50 mL of 10% and 40% methanol/water (*v*/*v*) sequentially. Finally, 0.15 mL of 70% methanol/water with 1% acetic acid was added, and the eluent was collected, diluted five times in water, and analyzed by LC-MS/MS.

### 5.4. LC-MS/MS Analysis

LC-MS/MS spectra were collected on an Agilent 1200 HPLC system (Palo Alto, CA, USA) coupled to an API 4000 triple quadrupole mass spectrometer (Applied Biosystems-SCIEX, Carlsbad, CA, USA). The ion source was a Turbo ion spray source operating in ESI^+^ mode. The LC system consisted of a G1322A vacuum degasser, a G1312A binary pump, a G1316A column compartment and a CTC autosampler (Leap Technology, Zwigen, Switzerland). Chromatographic separation was carried out on a Grace Alltima HP C18 (Grace Davison Discovery Sciences, Deerfield, IL, USA) (50 × 2.1 mm, 5 μm) column at ambient temperature. The LC elution conditions were as follows (all solvent percentages were by volume): mobile phase A, 2% methanol in water; mobile phase B, 0.1% formic acid in acetonitrile; gradient: 0 min, 100% A/0% B; 0.2 min, 100% A/0% B; 1.3 min, 50% A/50% B; 1.5 min, 10% A/90% B; 1.9 min, 10% A/90% B; 2.1 min, 100% A/0% B and 3 min, 100% A/0% B. The flow rate was 0.4 mL/min. All injection volumes were 10 μL.

The mass spectrometer was operated in the positive ion multiple reaction monitoring (MRM) mode. The ion source parameters were set as follows: curtain gas (nitrogen) = 30 p.s.i., temperature = 450 °C, gas 1 (nitrogen) = 50 p.s.i., gas 2 (nitrogen) = 50 p.s.i., ion spray voltage = 5500 V. The optimized parameters for the MRM analysis of GI with disulfide bridges were set as follows: transition (*m*/*z*):480.0→473.6, declustering potential (DP) = 40 V, collision energy (CE) = 19 eV. The optimized parameters for the MRM analysis of MI[ΔR2] with disulfide bridges were set as follows: transition (*m*/*z*):446.5→437.6, declustering potential (DP) = 50 V, collision energy (CE) = 20 eV.

### 5.5. Method Validation

The FDA guidelines for bioanalytical method validation were followed [35]. For specificity, six different batches of blank human plasma samples were analyzed. Plasma samples were pretreated as detailed in the sample preparation section. For linearity, seven different concentrations of GI in the range of 2.0–300.0 ng/mL in human plasma were analyzed. The ratios of GI area to IS area were plotted against the concentrations of GI in the plasma. The linear regression equation was obtained by least square fitting with a weighting factor of 1/*x*^2^. The correlation coefficient was reported. The lower limit of quantification (LLOQ) was chosen as the concentration of the lowest calibration standard if the analyte response at the LLOQ was at least ten times the response of the blank human plasma. Precision was evaluated as relative standard deviation (RSD%), while accuracy was calculated as relative error (RE%). The precision and accuracy of standard curves were determined by seven inter-batch plasma samples containing GI at the calibration curve standards, with six replicates for each standard. The inter-batch and intra-batch precision and accuracy were determined by analyzing QC samples at low, mid and high levels (6.0, 40.0 and 225.0 ng/mL) for all of the six different runs. The acceptance criterion for RSD% was within ±15% (±20% for LLOQ samples) and the criterion for RE% was within ±15 RSD% (±20% for LLOQ samples). The matrix interference on GI was evaluated by the spike recovery of GI at three QC levels spiked post extraction into the plasma extract against the corresponding peak areas of GI in the diluent. Matrix interference was considered negligible if the spike recovery was between 85% and 115%. The extraction recoveries of GI at three QC levels were determined by comparing the peak areas of the pre-extraction spiked plasma samples and those of the post-extraction spiked plasma samples. The stability of the QC samples was evaluated after sample storage at room temperature for 5.25 h, at 4 °C in the autosampler (pretreated) for 55 h, at −20 °C after three freeze/thaw cycles, and at −20 °C for 14 days (long term). The stability of the GI stock solutions was evaluated after sample storage at room temperature for 6 h (short term) and at 2–8 °C for 19 days (long term). The stability of the MI[ΔR2] stock solutions was evaluated after sample storage at room temperature for 6 h (short term) and at 2–8 °C for 23 days (long term).

### 5.6. Analysis of GI in Human Blood

The blood samples were collected from four individuals, each with two duplicate samples. A 40 μL aliquot of the GI working standard solution (5 μg/mL) was diluted in 1960 μL human blood and mixed slightly, resulting in a final GI concentration 100 ng/mL. The blood samples were incubated at 37 °C for 10 min to test recoveries and were incubated at room temperature for 3 h to test stabilities. The samples used for the recovery test were centrifuged at 3000 rpm to separate plasma. A 0.30 mL aliquot of the plasma samples was then processed as described in the “sample preparation” section and prepared for LC-MS/MS analysis. The mixed plasma was used in the preparation of standard curves.

### 5.7. Data Acquisition and Analysis

Data were processed using the Analyst (v1.6) software (Applied Biosystems-SCIEX, Carlsbad, CA, USA). Calibration curves were constructed by least square linear regression analysis using a weighting factor of 1/*x*^2^. Ratios of analyte peak areas versus IS peak area were calculated for each point.

## Figures and Tables

**Table 1:** The intra- batch precision and accuracy of GI at LLOQ.

Sample No.	Concentration (ng/mL)
	2.0
1	1.83
2	2.27
3	2.28
4	2.32
5	2.41
6	1.77
Mean (ng/mL)	2.15
SD	0.27
RSD%	12.70
RE%	7.27

SD: standard deviation; RSD%: relative standard deviation; RE%: relative error.

**Table 2:** The intra- and inter-batch precision and accuracy of GI QC samples.

Assay	Concentration (ng/mL)		
	6.0	40.0	225.0
Intra-batch (*n* = 6)			
Mean ± SD (ng/mL)	5.7 ± 0.3	40.5 ± 2.6	223.3 ± 9.1
RSD%	5.72	6.31	4.08
RE%	−4.80	1.25	−0.76
Inter-batch (*n* = 7)			
Mean ± SD (ng/mL)	6.1 ± 0.5	40.6 ± 2.9	227.9 ± 18.6
RSD%	8.61	7.02	8.16
RE%	1.47	1.50	1.27

SD: standard deviation; RSD%: relative standard deviation; RE%: relative error.

**Table 3:** Matrix effect and extraction recoveries of GI in plasma ^a^.

Concentration (ng/mL)	The Ratio of Peak Area ^b^ (A)	The Ratio of Peak Area ^c^ (B)	The Ratio of Peak Area ^d^ (C)	Matrix Effect ^e^ (%A)	Recovery ^f^ (%)
6.0	0.129	0.146	0.077	113.32(RSD%7.66)	52.81(RSD%2.53)
40.0	0.853	0.955	0.583	111.96(RSD%3.06)	61.07(RSD%9.19)
225.0	5.512	5.462	3.216	99.10(RSD%6.83)	58.88(RSD%3.90)
IS	1.173	1.048	0.700	89.36(RSD%2.36)	66.76(RSD%2.36)

^a^ *n* = 6; ^b^ The ratio of peak areas from samples; in pure water; ^c^ The ratio of peak areas from pre-extraction plasma samples; ^d^ The ratio of peak areas from post-extraction plasma samples; ^e^ Matrix effect(%) = (The mean ratio of peak areas from pre-extraction plasma samples)/(The mean ratio of peak areas from samples in pure water) × 100; ^f^ Extraction recovery(%) = (The mean ratio of peak areas from post-extraction plasma samples)/(The mean ratio of peak areas from pre-extraction plasma samples) × 100. The average recovery of GI was 57.59%. RSD%: relative standard deviation.

**Table 4:** Summary of stability of GI in human plasma.

Sample	QC1	QC2	QC3
Concentration	6.0 ng/mL	40.0 ng/mL	225.0 ng/mL
Room temperature (5.25 h)			
Mean concentration founded (*n* = 6)	5.9 ± 0.4	35.8 ± 2.5	200.5 ± 5.6
RSD%	6.58	7.00	2.81
RE%	−1.77	−10.54	−10.87
Autosampler(55 h)			
Mean concentration founded (*n* = 6)	6.2 ± 0.6	37.8 ± 4.3	203.5 ± 15.4
RSD%	9.11	11.37	7.54
RE%	3.13	−5.49	−9.56
Freeze-thaw (3-cycles)			
Mean concentration founded (*n* = 6)	5.6 ± 0.4	38.2 ± 4.0	205.7 ± 16.9
RSD%	6.77	10.38	8.21
RE%	−7.31	−4.53	−8.58
Long-term (14 days at −20 °C)			
Mean concentration founded (*n* = 6)	6.2 ± 0.6	42.0 ± 3.4	221.9 ± 12.6
RSD%	9.86	8.14	5.67
RE%	3.76	4.95	−1.38

RSD%: relative standard deviation; RE%: relative error.

**Table 5:** The analyses and recovery rates of GI in blood/plasma.

Matrix	Blood	Plasma
Mean volume founded (mL)		
*n* = 4	2.0	1.0
SD	0.00	0.07
RSD%	0.00	6.90
Mean concentration of GI (ng/mL)		
*n* = 4	100.0	141.5
SD	0.00	14.57
RSD%	0.00	10.30
Mean content of GI (ng)		
*n* = 4	200.0	147.0
SD	0.00	19.11
RSD%	0.00	13.00
The relative recovery of human blood (%) ^a^		
*n* = 4	73.48	
SD	9.55	
RSD%	13.00	
The absolute recovery of human blood (%) ^b^		
*n* = 4	42.32	
SD	5.50	
RSD%	13.00	

^a^ The relative recovery of human blood = (The amount of analyte in plasma/the amount of analyte in blood) × 100% = (the concentration of analyte in plasma × the volume of plasma)/(The theoretical concentration of analyte in blood × the volume of blood) × 100%; ^b^ The absolute recovery of human blood = the relative recovery of human blood × the recovery of plasma. SD: standard deviation; RSD%: relative standard deviation.
